# Supplementary figures and images for: Molecular Regulatory Network of Anthocyanin Accumulation in Black Radish Skin as Revealed by Transcriptome and Metabonome Analysis
Source: Int J Mol Sci. 2023 Sep 4;24(17):13663. doi: 10.3390/ijms241713663 (PMC10563070; doi:10.3390/ijms241713663)

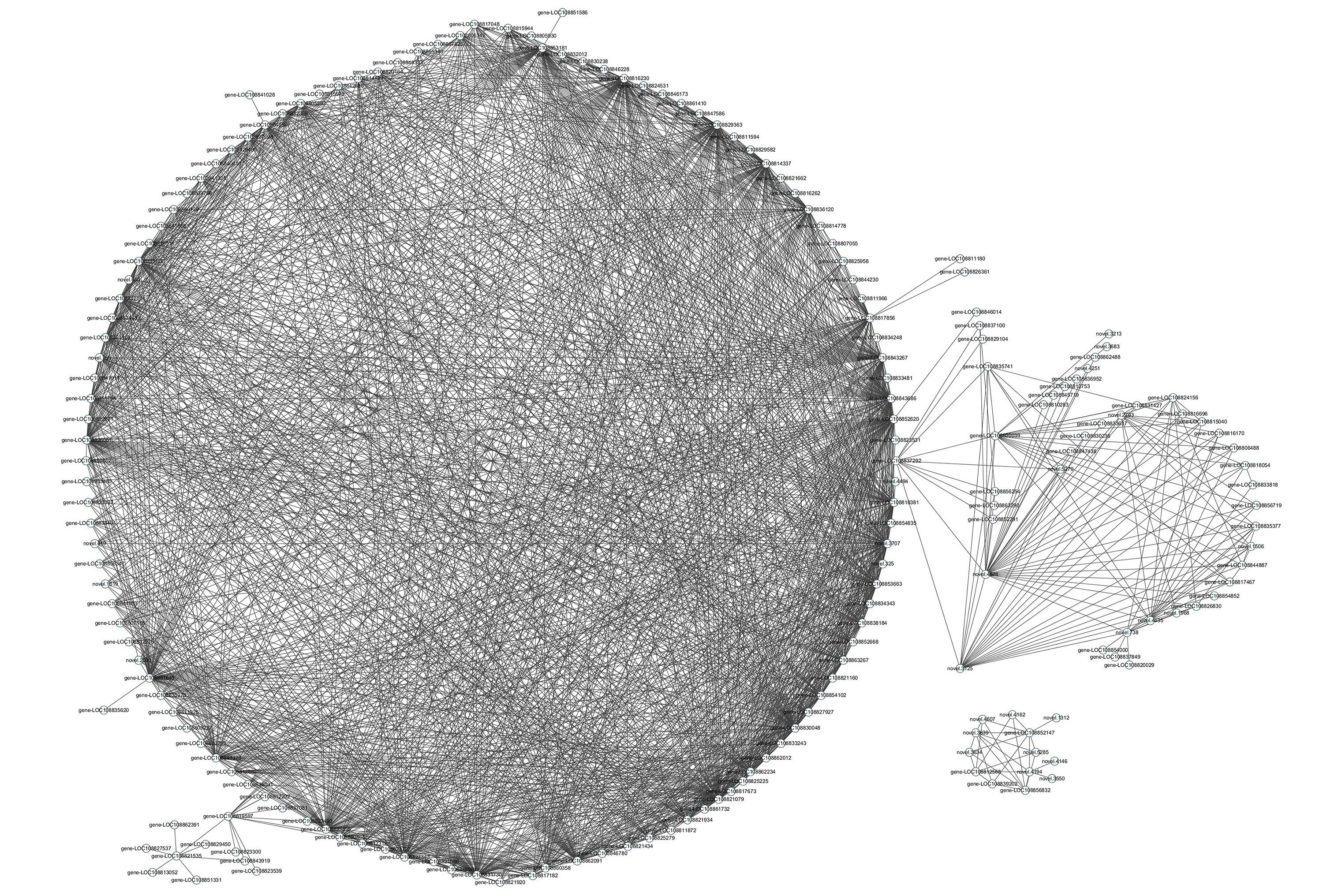

Supplement: Supplementary file 1 [file ijms-24-13663-s001.zip › Figure S1.jpg]
